# Agreement between automated and manual scoring of level 3 home sleep apnea devices: A single-center retrospective study

**DOI:** 10.1016/j.sleepx.2026.100193

**Published:** 2026-06-29

**Authors:** Sulaiman Khadadah, Hajar Alanazi, Yousra Saleh, Jabri Aljabri, Mohamed Fathi Elbagalaty, Anwar Ali, Mohammad Abdulsalam

**Affiliations:** a MKH Sleep and Neurophysiology Center, Mubarak Al Kabeer Hospital, Jabriya, Kuwait; b Department of Thoracic, Chest Diseases Hospital, Sabah, Kuwait; c College of Medicine, Kuwait University, Jabriya, Kuwait; d Chest Department, Faculty of Medicine, Ain Shams University, Cairo, Egypt

## Abstract

• Automated scoring on HSAT devices underestimate REI compared to manual scoring.
• Automated scoring underestimated OSA severity in up to 44.5% of moderate cases.
• Misclassification occurs near clinically relevant severity thresholds.
• Hypopnea index was significantly lower with automated compared to manual scoring.
• Findings highlight targets for improving automated and AI scoring.

## 1 Introduction

Obstructive sleep apnea (OSA) is a common sleep-related breathing disorder associated with adverse cardiovascular, metabolic, and neurocognitive outcomes [1]. Accurate diagnosis and appropriate severity classification are essential to guide treatment and reduce long-term morbidity [2]. While in-laboratory polysomnography (PSG) remains the diagnostic gold standard, Level 3 home sleep apnea testing (HSAT) has emerged as a widely used, cost-effective, and accessible alternative [2]. HSAT devices typically record nasal airflow, respiratory effort via respiratory inductance plethysmography (RIP), oxygen saturation (SpO_2_), heart rate, and body position, but usually lack electroencephalography channels, preventing the direct detection of arousals and total sleep time [2]. Consequently, respiratory events are indexed to recording time, producing a respiratory event index (REI) that is known to underestimate the PSG-derived apnea–hypopnea index (PSG-AHI) [2], one of several recognized limitations of event-count severity indices [3] that are further compounded by substantial night-to-night variability [4].

Manual scoring of HSAT data by trained technologists, using the American Academy of Sleep Medicine (AASM) criteria, remains the reference standard for interpreting HSAT studies. However, manual reviews are time-consuming and resource intensive. HSAT built-in automated scoring algorithms are increasingly used to address the growing demand for sleep studies. Whether these automated algorithms reliably reproduce manual scoring at clinically meaningful levels of accuracy remains uncertain: prior comparative studies have reported conflicting results, were typically performed in small samples, and have varied in device platform, scoring algorithm, and analytic methodology [5].

Even when performed by an expert technologist using AASM criteria, manual HSAT scoring underestimates the true burden of disease, since HSAT lacks EEG and therefore cannot detect hypopneas terminated by cortical arousals alone. To overcome this limitation, the concept of autonomic hypopnea (AnH) was introduced [6]. AnHs are respiratory events meeting hypopnea airflow criteria accompanied by a heart rate acceleration of ≥6 bpm, as measured from the oximeter pulse waveform and correlate with arousal-related hypopneas [7]. Manual HSAT scoring that includes AnH events yields an REI that more closely approximates the PSG-AHI [7,8], and therefore provides a closer approximation of OSA severity than standard HSAT scoring in the absence of in-laboratory PSG.

The primary objective of this study was to assess the agreement between manufacturer-automated and expert manual scoring of HSAT-derived REI in a large real-world clinical cohort, using Bland–Altman analysis to quantify systematic bias, and reclassification analysis to determine the clinical impact on OSA severity at the individual-patient level. We hypothesized that automated scoring would systematically underestimate the manually scored REI, with sufficient bias to misclassify OSA severity in a clinically meaningful proportion of patients. Manual scoring incorporating AnH served as the pragmatic reference standard in the absence of in-laboratory PSG.

## 2 Patients and methods

### 2.1 Study design and setting

This single-center retrospective observational study was conducted at the Sleep Clinic of Mubarak Al-Kabeer Hospital, Kuwait. Adults referred for evaluation of suspected OSA between January 2021, and June 2023 were included.

### 2.2 Ethical considerations

The study protocol was approved by the institutional review board of the Kuwait Ministry of Health (2025/2930). As the study involved retrospective analysis of clinically acquired data with no direct patient contact, informed consent was waived in accordance with institutional policy.

### 2.3 Participants

Studies were included if HSAT recordings were of adequate technical quality. Adequate technical quality was defined as a minimum recording duration of ≥4 h with ≥95% valid signal on each of the primary channels (nasal pressure airflow and pulse oximetry). Exclusion criteria included severe cardiopulmonary disease, severe psychiatric illness, or inability to correctly apply the HSAT device despite instructions.

### 2.4 HSAT acquisition

Overnight level 3 HSATs were performed using the SOMNOtouch device (SOMNOmedics GmbH, Randersacker, Germany) following the 2023 AASM standard montage recommendations. The device recorded nasal airflow (pressure transducer), thoracic effort RIP (inductance belt), SpO_2_ and pulse rate (sampled at 75 Hz with a four-beat averaging time), snoring, and body position. All patients or caregivers received in-person training from a technologist and were provided with written and video instructions.

### 2.5 Scoring approaches

Each HSAT study was evaluated using three predefined scoring approaches:

1. Automated scoring: The manufacturer's proprietary algorithm generated an automated respiratory event index. The REI was obtained and labeled as the Automated REI.
2. Manual AASM scoring (standard): Manual scoring was performed by an experienced registered PSG technologist (RPSGT) using a standardized scoring protocol with AASM criteria. Scoring was done according to the AASM 2023 rules:
  ￮ Apnea: ≥90% reduction in airflow from baseline for ≥10 s.
  ￮ Hypopnea: ≥30% reduction in airflow from baseline for ≥10 s with an associated ≥3% oxygen desaturation.
  ￮ Results labeled as standard REI (AASM-defined apneas and hypopneas only).
  ￮ Manual scoring was performed independently by a trained RPSGT using raw data, with the automated scoring output hidden from view during manual scoring to prevent any bias.
3. Manual AASM scoring with AnH (extended): Manual AASM scoring supplemented by the identification of airflow reduction events meeting hypopnea airflow criteria accompanied by a pulse oximetry-derived heart rate acceleration of ≥6 bpm (AnH) [6,8]. Result labeled as extended REI.

### 2.6 Statistical analysis

All statistical analyses were conducted using a two-sided significance and an alpha level of 0.05. Continuous variables are summarized as mean ± standard deviation (SD). Paired t-tests were used to compare REI, oxygen desaturation index (ODI), apnea index (AI), and hypopnea index (HI) between the automated and manual scoring methods. Agreement between the scoring methods was assessed using Bland–Altman analysis to quantify the bias (mean difference) for the REI (standard and extended), AI, HI, and ODI; 95% limits of agreement are reported for the REI comparisons. Pearson correlation coefficients were calculated to assess the linear associations between the automated and manual REI (both standard and extended REI).

For the OSA severity classification, we generated cross-tabulations of the automated and manual scoring outcomes. Cohen's kappa statistics were calculated to quantify the overall agreement in the categorical severity assignments between automated and manual scoring. Given that OSA severity is defined by an ordinal value (REI cutoff), we also computed weighted kappa coefficients when comparing automated scoring with the extended REI. OSA severity was classified using standard REI cutoffs as follows: normal (no OSA; <5 events/h), mild (≥5 to <15 events/h), moderate (≥15 to <30 events/h), and severe (≥30 events/h). Kappa coefficients are reported with 95% confidence intervals (CIs) where applicable. Finally, a reclassification analysis was performed to determine the proportion of patients whose OSA severity category changed under automated scoring, using the extended and standard manual REI as references. All analyses were performed using the R statistical software, and the results were reviewed for clinical and statistical significance. Methods and results are reported in accordance with STROBE guidelines.

## 3 Results

### 3.1 Demographic characteristics

A total of 1026 patients who underwent technically adequate HSAT were included in the analysis. The mean age was 49.9 ± 17.9 years, the mean body mass index was 38.3 ± 9.3 kg/m^2^, and 60.3% (n = 619) were male.

### 3.2 Comparison of automated and manual REI

**Fig. 1:**
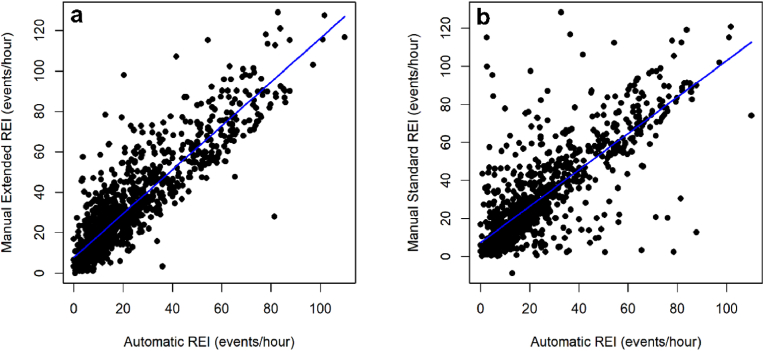
(a) Correlation between the automated and manual extended REI. (b) Correlation between the automated and manual standard REI. REI, respiratory event index.

Automated REI was strongly correlated with both manual scores (extended REI, r = 0.755 [95% CI 0.727–0.780; Fig. 1a]; standard REI, r = 0.780 [95% CI 0.755–0.803; Fig. 1b]). Despite these strong correlations, Bland–Altman analysis demonstrated systematic between-method differences.

**Fig. 2:**
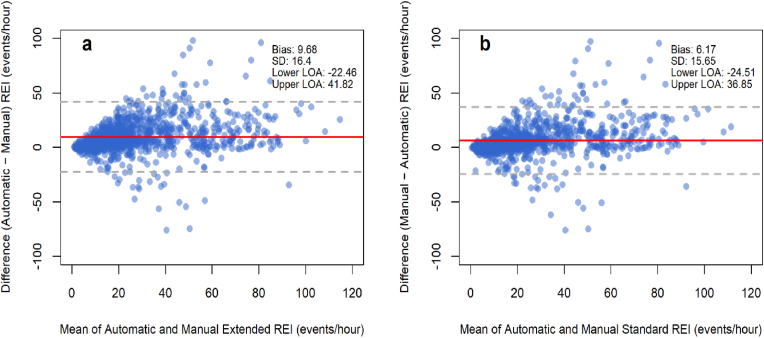
Bland–Altman analysis. (a) Extended REI vs. automated REI. (b) Standard REI vs. automated REI. REI, respiratory event index; LOA, limit of agreement; SD, standard deviation.

For extended REI, the mean difference (manual minus automated) was 9.7 ± 16.4 events/h, corresponding to 95% limits of agreement of −22.5 to 41.8 events/h. These wide limits indicate substantial variability at the individual-patient level. These differences tended to increase with higher event rates. The direction of bias was consistently positive (manual minus automated), indicating that automated scoring yielded lower REI values than manual scoring across the range of measurements (Fig. 2a).

When manual scoring was restricted to standard AASM criteria (standard REI), the mean difference decreased to 6.2 ± 15.7 events/h (95% limits of agreement, −24.5 to 36.9 events/h); however, the limits of agreement remained wide (Fig. 2b). These findings indicate that, although automated and manual REI values are statistically correlated, variability between scoring methods persists at the individual patient level, with automated scoring generally yielding lower REI values.

### 3.3 Impact of AnH

**Fig. 3:**
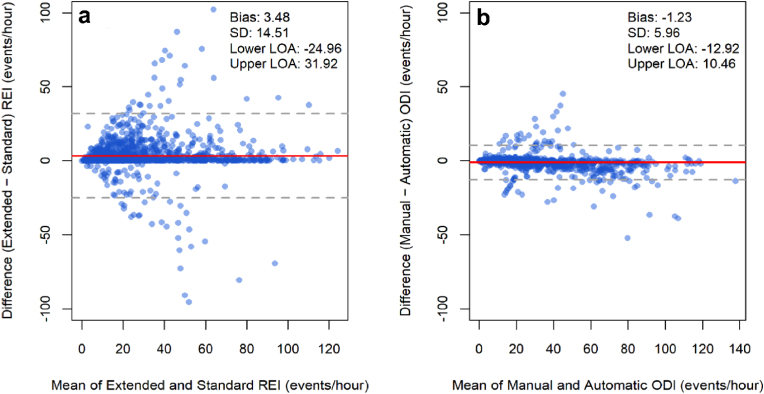
a & b; Comparison of extended and standard REI and ODI for manual and automated scoring. (a) Bland–Altman analysis of extended vs. standard REI. (b) Bland–Altman analysis of automated and manual scoring for ODI. LOA, limit of agreement; REI, respiratory event index; ODI, oxygen desaturation index.

Extended manual scoring, which included AnHs, yielded higher REI values than standard manual scoring. The mean difference between extended REI and standard REI was +3.4 events/h, resulting in upward reclassification of OSA severity in a subset of patients. The inclusion of AnHs increased the estimated REI values without altering the ODI measurements (Fig. 3a and b).

### 3.4 OSA severity classification and agreement

**Fig. 4:**
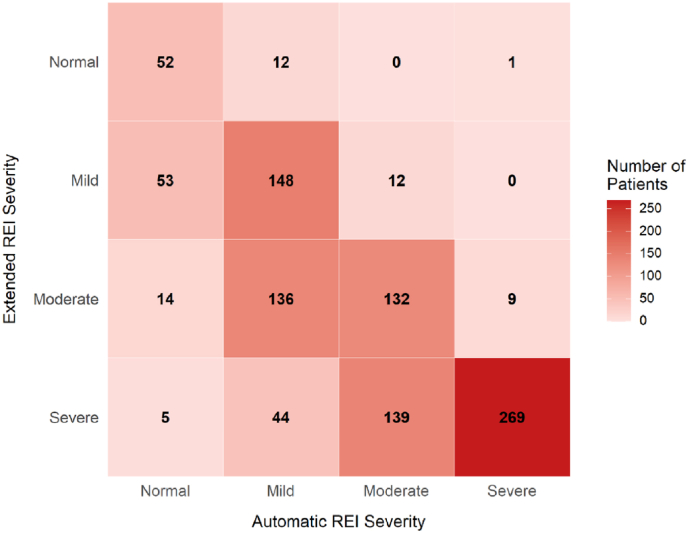
Obstructive sleep apnea diagnosis classification agreement matrix for automated REI and extended REI. REI, respiratory event index.

Using the manually extended REI as the reference for severity classification and reclassification, OSA was identified in 93.7% of patients, compared with 87.9% using automated scoring. Automated scoring more frequently assigned lower-severity categories relative to the extended REI, particularly in patients with moderate and severe OSA. Specifically, automated scoring underestimated OSA severity in 24.9% of patients classified as mild, 51.5% of those classified as moderate, and 41.1% of those classified as severe by the extended REI (Fig. 4).

Automated and manual extended scoring assigned the same OSA severity category in 58.6% of patients (unweighted Cohen's kappa, 0.43; 95% CI, 0.39–0.47). Among the patients classified differently, 92% were assigned a lower severity category by automated scoring and 85% differed by a single category (Fig. 4). The linear-weighted kappa was 0.57 and the quadratic-weighted kappa was 0.69, the higher values reflecting that most disagreements spanned only one severity category.

**Fig. 5:**
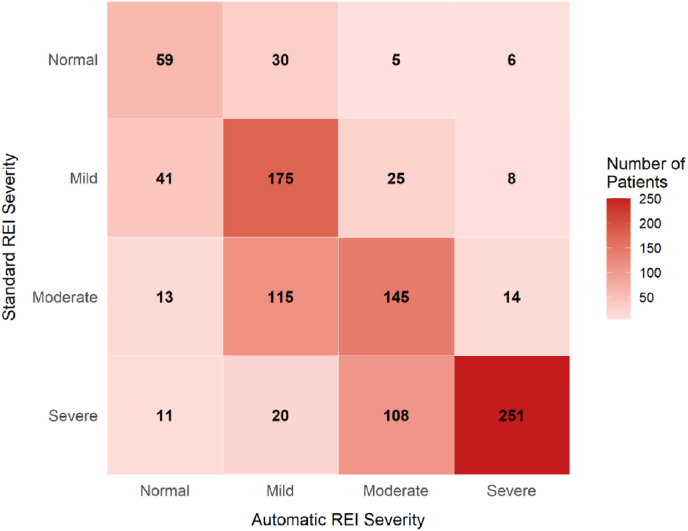
Obstructive sleep apnea diagnosis classification agreement matrix for automated REI and standard REI. REI, respiratory event index.

Against the manual standard REI, the two methods assigned the same severity category in 61.4% of patients (unweighted Cohen's kappa, 0.47). The disagreement was again directional: among patients classified differently, 78% were assigned a lower category by automated scoring and 84% differed by a single category. Automated scoring underestimated severity in 16.5% of mild, 44.6% of moderate, and 35.6% of severe OSA cases relative to standard manual scoring (Fig. 5). Although the exclusion of AnHs reduced numerical differences between methods, the limits of agreement for the standard REI remained wide, and automated scoring continued to assign lower severity categories to a substantial proportion of patients.

### 3.5 ODI, HI, and AI

The ODI demonstrated close agreement between automated and manual scoring. Mean ODI values did not differ significantly between methods (manual: 28.5 ± 24.3 vs. automated: 29.7 ± 26.6 events/h; p = 0.27; Table 1) and Bland–Altman analysis showed minimal bias (Fig. 3b).

**Table 1:** Sleep parameter data detected by manual and automated scoring among studied patients (n = 1026).

Sleep Parameter Data	Manual Score (Mean ± SD)	Automated Score (Mean ± SD)	Significance
REI (Standard)	29.7 ± 25.0	23.5 ± 20.3	p < 0.001
REI (Extended)	33.1 ± 24.9	23.5 ± 20.3	p < 0.001
AI	12.0 ± 18.6	10.4 ± 11.3	p < 0.001
HI (Standard)	17.8 ± 15.2	12.4 ± 12.3	p < 0.001
HI (Extended)	21.0 ± 15.3	12.4 ± 12.3	p < 0.001
ODI	28.5 ± 24.3	29.7 ± 26.6	p = 0.270
TRT (hours)	6.13 ± 1.87	—	—

REI, respiratory event index; AI, apnea index; HI, hypopnea index; ODI, oxygen desaturation index; TRT, total recording time; SD, standard deviation. Manual values labeled “Standard” use AASM criteria (apneas plus desaturation-based hypopneas); values labeled “Extended” additionally include autonomic hypopneas (AnH). The Significance column reports paired *t*-test statistics comparing manual versus automated scoring.

In contrast, HI differed substantially between the scoring methods. Both manual HI values were consistently higher than automated HI values (standard manual HI 17.8 ± 15.2 and extended manual HI 21.0 ± 15.3 vs. automated 12.4 ± 12.3 events/h; both p < 0.001), a mean difference of 5.4 and 8.6 events/h, respectively, indicating that variability in hypopnea detection between methods was a major contributor to differences in REI (Table 1). The AI differed statistically between automated and manual scoring (manual: 12.0 ± 18.6 vs. automated: 10.4 ± 11.3 events/h; p < 0.001; Table 1). However, Bland–Altman analysis revealed a small mean difference of only +1.6 events/h, substantially smaller than the bias observed for the hypopnea index or the REI. This suggests that the AI difference, although statistically significant owing to the large sample size, is unlikely to be clinically meaningful, and that the disparity in REI was driven predominantly by hypopnea detection rather than apnea detection. The mean total recording time was 6.13 ± 1.87 h.

## 4 Discussion

This study examined the agreement between automated and manual HSAT scores in a large cohort of patients with suspected OSA. Although automated scoring showed strong statistical correlations with manual scoring at the group level, the reclassification analysis demonstrated that the two scoring methods can result in significant discrepancies in the REI for OSA severity and diagnosis. The Bland–Altman analysis revealed wide limits of agreement and a consistent positive bias (manual minus automated), indicating a systematic underestimation of the REI by automated scoring across the range of OSA severities. The observed bias was of sufficient magnitude to cross the established OSA severity thresholds, which explains the high rate of severity reclassification observed in our cohort. These findings underscore that despite the strong statistical correlation between automated and manual scoring; automated scoring does not equate to an acceptable clinical agreement and may be less reliable for severity classification in patients near clinically relevant thresholds [4]. Notably, the Pearson coefficient reflects linear association rather than absolute agreement: although both manual scores correlated strongly with automated scoring, Bland–Altman analysis revealed wide limits of agreement and a systematic bias. This dissociation between strong correlation and poor agreement is precisely why Bland–Altman and reclassification analyses, rather than correlation alone, are needed to judge clinical interchangeability. This downward misclassification may delay treatment initiation or under-recognize disease severity, particularly for patients whose automated REI values fall just below diagnostic or therapeutic thresholds [8].

Although ODI and AI showed strong concordance between methods, the largest difference was seen in REI and HI values for manual versus automated scoring, suggesting the limited sensitivity of automated algorithms for hypopnea detection. The inclusion of AnHs increased the manually derived REI values and resulted in the upward reclassification of disease severity in a subset of patients. However, excluding AnHs did not resolve the discrepancy between the scoring methods. This indicates that systematic misclassification is not solely attributable to AnH inclusion and reflects the broader limitations of automated event detection in HSAT. In our cohort, the disagreement between automated and manual scoring was more pronounced in patients with moderate and severe OSA.

Of note, only studies meeting predefined technical adequacy criteria (≥4 h of recording with ≥95% valid signal on each primary channel) were included in the present analysis; the systematic automated underestimation of REI and HI was therefore observed in studies with acceptable signal quality. Algorithm performance may be expected to degrade further in studies with marginal signal quality, which were excluded here by design.

In the absence of PSG as the gold-standard comparison for OSA severity, we used the extended REI as a pragmatic approximation of the PSG-AHI. The AnH concept, previously introduced and validated against PSG [[6], [7], [8]], uses heart rate acceleration as a surrogate for cortical arousal to capture hypopneas that would otherwise be missed by conventional AASM scoring. The present analysis applied AnH scoring in what is, to our knowledge, the largest cohort to date (n = 1026). As expected from prior studies, the inclusion of AnH increased the manual REI relative to standard AASM scoring. Importantly, automated scoring underestimated the manual reference regardless of whether the standard or the extended REI was used as the comparator, indicating that the observed discrepancy between automated and manual scoring is not an artifact of AnH inclusion. Moreover, comparing automated scoring against the extended REI reveals that the OSA severity reclassification produced by automated scoring is even further from the severity that PSG would be expected to capture than a comparison against the standard manual REI alone would indicate.

Our findings align with those of previous studies that directly compared automated and technician-measured manual HSAT scoring [[9], [10], [11]]. These studies reported variable agreements between automated systems and manual scoring across international centers, emphasizing that automated outputs may not reliably match technician scoring in all cases. Another study found that automated HSAT analysis, similar to our study, could underestimate the REI and alter the OSA severity classification, supporting the clinical relevance of the severity shifts observed in our study [11]. Our analysis extends these prior reports to a substantially larger, real-world cohort and, in the absence of in-laboratory PSG, uses the extended REI as a pragmatic approximation of the PSG-AHI to approximate the burden of OSA that PSG would capture [8].

In contrast to our findings, Magalang et al. reported closer agreement between automated and technician manual HSAT scoring, with narrower limits of agreement and higher concordance metrics than those observed in our cohort [12]. Several design factors likely explain this divergence. Magalang et al. analyzed 15 HSAT studies across nine technologists and two commercial algorithms, whereas our analysis included 1026 consecutive studies using a single device, a single algorithm, and a single experienced scorer. Multi-algorithm and multi-scorer designs introduce variability from different algorithms and inter-scorer differences that can mask algorithm specific bias or performance; in contrast our single algorithm, single scorer design isolates the algorithm-versus-manual difference, and combined with a substantially larger sample, provides greater statistical power to detect patient-level severity reclassification. Together, these observations suggest that automated HSAT scoring may be insufficiently accurate for stand-alone clinical use, and its accuracy may be further affected by device, algorithm, signal quality, and patient cohort [5].

Looking forward, advances in artificial intelligence and machine-learning–based approaches show promise in bridging the gap between manual and automated scoring; however, studies reporting the equivalence or superiority of automated methods have largely relied on in-laboratory PSG or clinical feature–based prediction models rather than HSAT signal-based scoring. As such, these findings are not directly comparable to those of the present study, which focused on HSAT performance. Nevertheless, machine-learning–based scoring algorithms continue to evolve rapidly, and ongoing developments incorporating multiple signals and large real-world datasets may improve the accuracy and clinical reliability of automated HSAT interpretation in the future [13].

More broadly, the present study, like most of the existing OSA diagnostic literature, relies on event-count indices (REI, HI, and ODI). It is increasingly recognized that these indices have important limitations, and the scoring-method discrepancies we report represent one further such limitation. New complementary measures of OSA severity including hypoxic burden [14], symptom-based phenotypes such as the excessively sleepy subtype [15], excessive daytime sleepiness, and comorbidity burden have been proposed and validated as more clinically meaningful descriptors of disease severity and prognosis. Incorporating these parameters alongside, or in place of, event counts may mitigate some of the limitations inherent to event-count–based interpretation of HSAT.

This study had several limitations that merit consideration. First, PSG was not performed; therefore, the diagnostic accuracy relative to that of a level 1 sleep study could not be directly assessed. Instead, an extended REI incorporating AnHs was used as a pragmatic surrogate to estimate true OSA severity, which may not fully replicate the PSG-AHI in all patients. Although the extended REI has been previously validated as a closer approximation to PSG-AHI than standard HSAT scoring [8], the precise direction and magnitude of any residual discrepancy against true PSG-AHI cannot be fully characterized in the absence of simultaneous PSG. Second, the study was conducted at a single center, which may limit its generalizability to other clinical settings. Third, although manual scoring was performed independently by an experienced RPSGT to ensure internal consistency, intra-scorer variability was not evaluated and could influence manual scoring outcomes. However, we used standard scoring methods. Fourth, the study population had a relatively high mean body mass index and consisted of patients referred for suspected OSA, which may limit the extrapolation of these findings to lower-risk or asymptomatic populations. Finally, the proprietary details of the automated scoring algorithm were not available, preventing the evaluation of specific signal processing or event detection components responsible for the observed discrepancies. Despite these limitations, the large sample size and real-world clinical setting provide useful insight into the performance of automated versus manual HSAT scoring in routine practice.

In summary, automated HSAT scoring, although efficient to perform, systematically underestimates OSA severity compared with manual scoring. This underestimation is further exaggerated when compared with the extended manual REI, which incorporates AnH as a pragmatic approximation of the PSG-AHI. Until automated algorithms demonstrate consistent accuracy across severity thresholds and patient populations, expert manual review remains important to ensure accurate diagnosis and appropriate clinical management.

## Consent to participate

As the study involved a retrospective analysis of clinically acquired data with no direct patient contact, informed consent was waived in accordance with institutional policy.

## Ethics approval

The study protocol was approved by the Institutional Review Board of the Kuwait Ministry of Health (approval number: 2026/3199), and was performed in accordance with the ethical standards as laid down in the 1964 Declaration of Helsinki and its later amendments or comparable ethical standards.

## Consent for publication

Not Applicable.

## Funding

No funding was received for this study.

## CRediT authorship contribution statement

**Sulaiman Khadadah:** Conceptualization, Data curation, Formal analysis, Investigation, Methodology, Project administration, Writing – original draft, Writing – review & editing. **Hajar Alanazi:** Data curation, Formal analysis. **Yousra Saleh:** Data curation, Formal analysis. **Jabri Aljabri:** Data curation. **Mohamed Fathi Elbagalaty:** Conceptualization, Data curation, Formal analysis, Project administration. **Anwar Ali:** Data curation, Validation, Writing – review & editing. **Mohammad Abdulsalam:** Conceptualization, Data curation, Methodology, Project administration, Writing – review & editing.

## Declaration of competing interest

The authors declare that they have no known competing financial interests or personal relationships that could have appeared to influence the work reported in this paper.

## Data Availability

The datasets generated and analyzed during the current study are not publicly available due to ethical and institutional restrictions related to patient confidentiality. De-identified data may be made available from the corresponding author upon reasonable request and subject to appropriate institutional approvals.
